# Shikonin reduces M2 macrophage population in ovarian cancer by repressing exosome production and the exosomal galectin 3-mediated β-catenin activation

**DOI:** 10.1186/s13048-024-01430-3

**Published:** 2024-05-14

**Authors:** Min Wang, Yangyan Sun, Rui Gu, Yan Tang, Guorong Han, Shaojie Zhao

**Affiliations:** 1 Department of Gynaecology and Obstetrics, The Second Hospital of Nanjing, Nanjing University of Chinese Medicine, No.1, Zhongfu Road, Nanjing, Jiangsu 210003 P.R. China; 2 Department of Gynaecology, Wuxi Maternity and Child Health Care Hospital, Affiliated Women’s Hospital of Jiangnan University, No. 48, Huaishu Lane, Liangxi District, Wuxi, Jiangsu 214000 P.R. China; 3 Department of Gynecology, Jiangyin People’s Hospital, Wuxi, Jiangsu 214400 P.R. China

**Keywords:** Shikonin, Galectin 3, β-catenin, Exosomes, M2 macrophages, Ovarian cancer

## Abstract

**Background:**

Shikonin (SK), a naphthoquinone with anti-tumor effects, has been found to decrease production of tumor-associated exosomes (exo). This study aims to verify the treatment effect of SK on ovarian cancer (OC) cells, especially on the production of exo and their subsequent effect on macrophage polarization.

**Methods:**

OC cells SKOV3 and A2780 were treated with SK. The exo were isolated from OC cells with or without SK treatment, termed OC exo and SK OC exo, respectively. These exo were used to treat PMA-induced THP-1 cells (M0 macrophages). M2 polarization of macrophages was determined by measuring the M2 specific cell surface markers CD163 and CD206 as well as the secretion of M2 cytokine IL-10. The functions of galectin 3 (LGALS3/GAL3) and β-catenin in macrophage polarization were determined by gain- or loss-of-function assays. CB-17 SCID mice were subcutaneously injected with SKOV3 cells to generate xenograft tumors, followed by OC exo or SK OC exo treatment for in vivo experiments.

**Results:**

SK suppressed viability, migration and invasion, and apoptosis resistance of OC cells in vitro. Compared to OC exo, SK OC exo reduced the M2 polarization of macrophages. Regarding the mechanism, SK reduced exo production in cancer cells, and it decreased the protein level of GAL3 in exo and recipient macrophages, leading to decreased β-catenin activation. M2 polarization of macrophages was restored by LGALS3 overexpression but decreased again by the β-catenin inhibitor FH535. Compared to OC exo, the SK OC exo treatment reduced the xenograft tumor growth in mice, and it decreased the M2 macrophage infiltration within tumor tissues.

**Conclusion:**

This study suggests that SK reduces M2 macrophage population in OC by repressing exo production and blocking exosomal GAL3-mediated β-catenin activation.

**Supplementary Information:**

The online version contains supplementary material available at 10.1186/s13048-024-01430-3.

## Background

While breast cancer is often the first that comes to the mind when mentioning fatal gynecologic malignancies, it is essential to recognize that ovarian cancer (OC) holds the distinction of being the most lethal one [1]. The 2020 global cancer statistics suggests a total of 313,919 newly diagnosed OC cases in the year, and 207,252 individuals died from it [2]. Epithelial cases constitute around 90% of OC instances, with serous carcinoma being the most prevalent subtype [3]. Unfortunately, approximately 70% of diagnoses occur at advanced stages due to the nonspecific signs or symptoms of ovarian tumors [4]. The 5-year survival rate for patients in advanced stages is merely around 30%, dropping to 15% at the 10-year mark [5]. Presently, the standard therapy for newly diagnosed advanced OC patients involves cytoreductive surgery and platinum-based chemotherapy; however, this approach is not without challenges, as roughly 70% of patients experience a relapse within three years, and the condition is typically incurable [6]. The severe situation challenges clinicians and scientists to discover new therapeutic options.

The growing interest in natural products with potential anticancer properties is attributed to their favorable safety and effectiveness [7]. Shikonin (SK, 5,8-dihydroxy-2-[(1R) − 1‑hydroxy-4-methylpent-3-enyl] naphthalene-1,4‑dione), a major component of the traditional Chinese medicine *Lithospermum erythrorhizon* (Purple Cromwell), has shown therapeutic promise in treating various conditions, like wound healing, inflammation, and cancer [8, 9]. SK has been summarized to possess a favorable therapeutic effect on cancer growth, development, and metastasis by regulating cancer cell proliferation, apoptosis, autophagy, necroptosis, and inducing immunogenic cell death [10]. In OC, SK has been found to subdue viability while promoting apoptosis of SKOV3 and A2780 cells [11, 12]. More interestingly, SK has been reported to reduce the release of exosomes (exo) from breast cancer MCF-7 cells to reduce cell proliferation [13]. Exo are small membrane vesicles with a diameter of 30–100 nm secreted by various types of cells [14, 15]. They can carry cell-specific cargoes of proteins, lipids, and genetic materials, and can be selectively taken up by neighboring or distant cells, reprogramming the recipient cells through the delivery bioactive compounds [16]. In cancer biology, exo are enriched in the tumor microenvironment (TME) and play a pivotal role in local and systemic cell-cell communication and regulate carcinogenesis, angiogenesis, proliferation, metastasis, and immunosuppression [17]. Macrophages, the most abundant immune cells in the TME [18], are briefly categorized into tumor-suppressive M1 phenotype and tumor-promoting M2 phenotype, with the latter form existing as the predominant population in the TME that inhibits anti-tumor immune response and favors tumor progression [19]. In this study, our objective is to explore the impact of SK treatment on OC cells, particularly on its effect on the secretion of OC cell-derived exo and their influence on M2 macrophages, along with the underlying molecular mechanisms.

## Results

### SK treatment suppresses malignant phenotype of OC cell lines

**Fig. 1:**
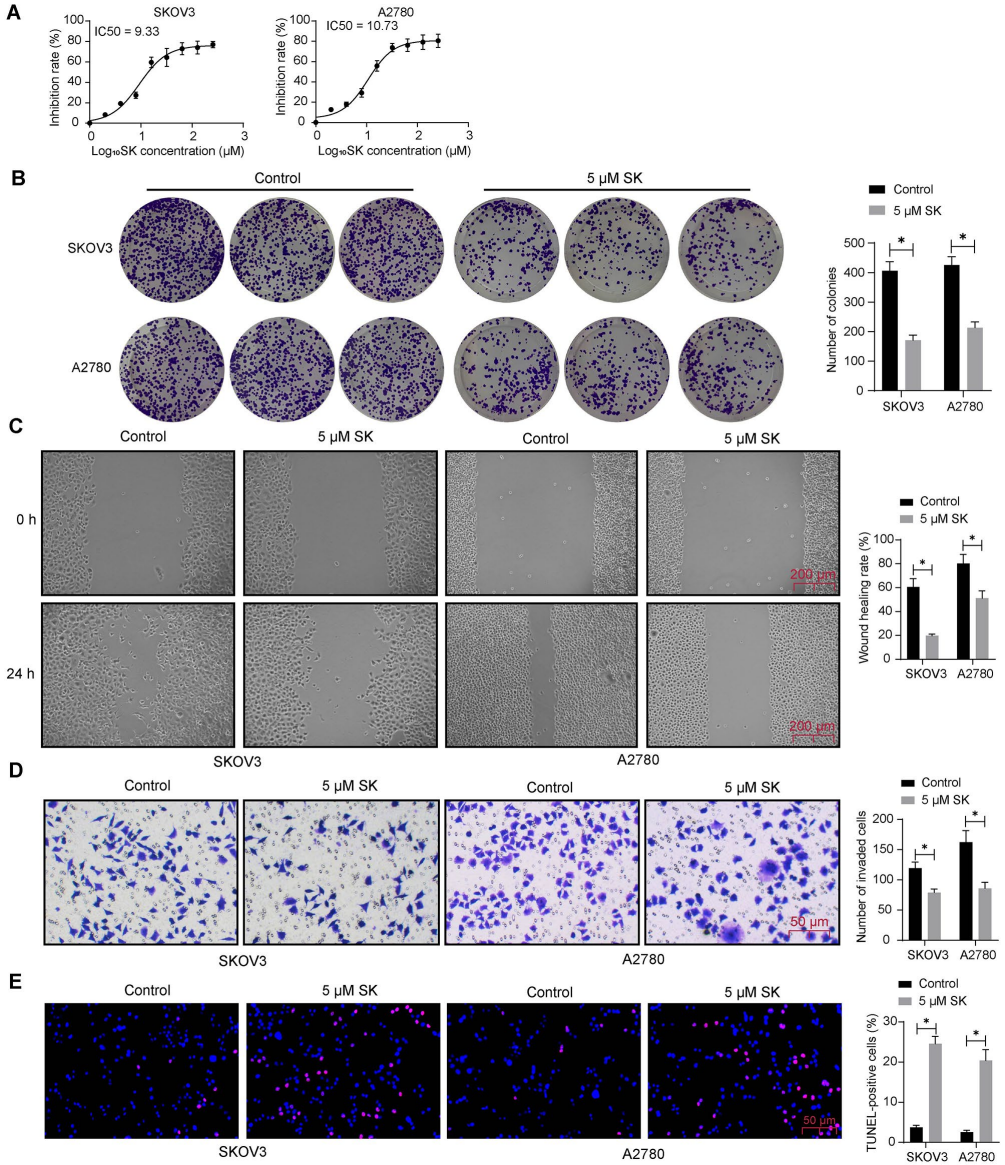
SK treatment suppresses malignant phenotype of OC cell lines. **A**, IC50 value of SK to SKOV3 and A2780 cells tested by applying different doses of SK (1 µM, 2 µM, 4 µM, 8 µM, 16 µM, 32 µM, 64 µM, 128 µM, and 256 µM) for 48 h, examined by CCK-8 assay. SKOV3 and A2780 cells were treated with 5 µM SK for 48 h. **B**, colony formation capacity of SKOV3 and A2780 cells determined by colony formation assay; **C**, migration ability of cells analyzed by wound healing assay; **D**, invasion ability of cells determined by Transwell assay; **E**, apoptosis of cells measured by TUNEL assay. Three biological replicates were performed. Differences were analyzed by two-way ANOVA. *p < 0.05

To evaluate the treatment efficacy of SK on OC, the SKOV3 and A2780 cells were treated with ascending series doses (1 µM, 2 µM, 4 µM, 8 µM, 16 µM, 32 µM, 64 µM, 128 µM, and 256 µM) of SK for 48 h. The CCK-8 assay concerning viability of both cell lines revealed an IC50 value of SK at 9.33 µM to SKOV3 cells and 10.73 µM to A2780 cells (Fig. 1A). A fixed dose of 5 µM was applied for subsequent analysis. This treatment significantly reduced the colony formation capacity of SKOV3 and A2780 cells (Fig. 1B) (Supplementary Fig S1A). In addition, the SK suppressed migration and invasion abilities of the OC cells according to the wound healing and Transwell assays, respectively (Fig. 1C-D). When it comes to cell apoptosis, TUNEL results revealed an increase in TUNEL positive rate in cells after the SK treatment (Fig. 1E), indicating increased cell apoptosis.

### SK treatment alleviates the promoting effect of OC cell-derived exo on M2 polarization of macrophages

**Fig. 2:**
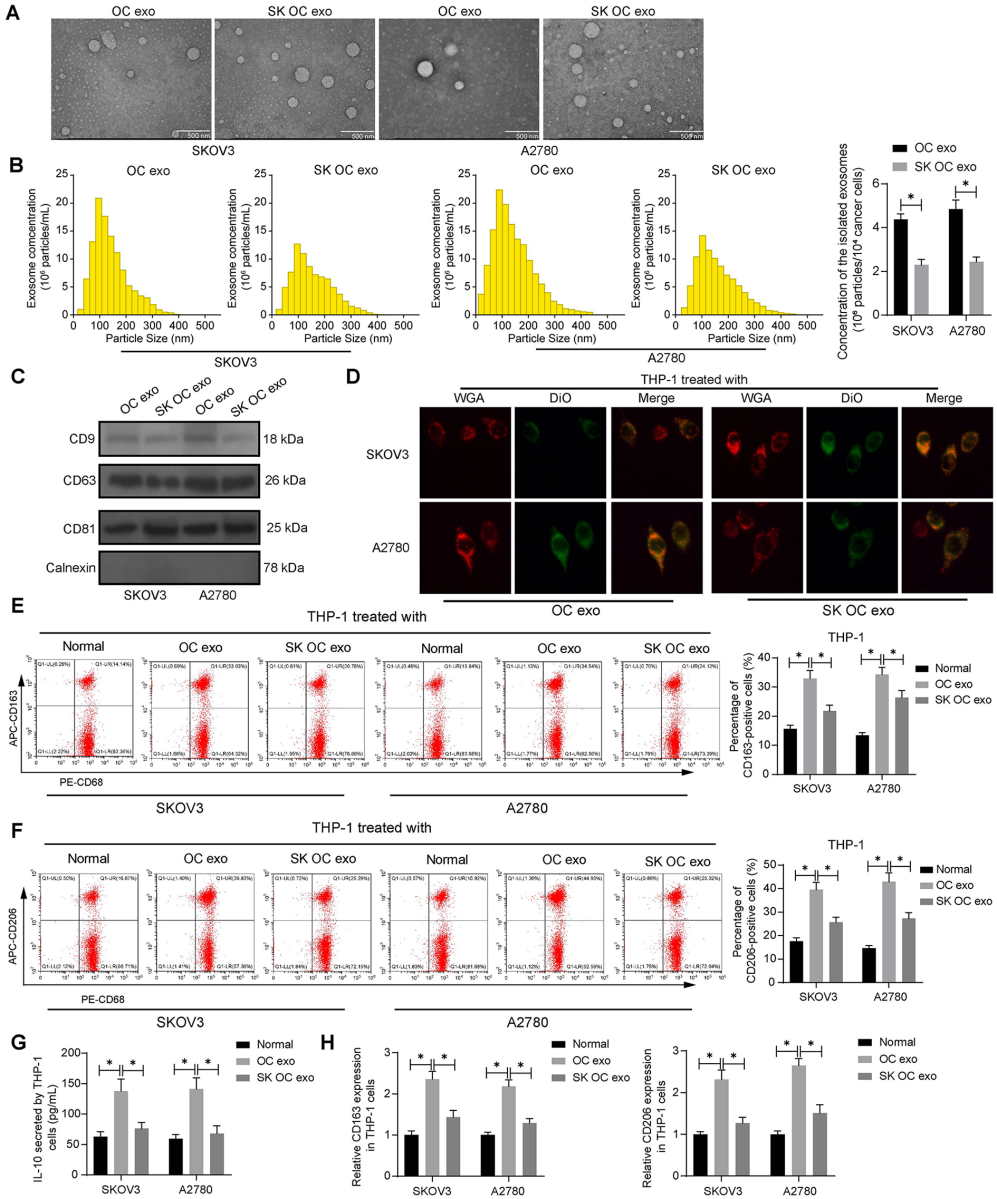
SK treatment reduces the promoting effect of OC cell-derived exo on M2 polarization of macrophages. Exo from SKOV3 and A2780 cells, either with or without 5 µM SK treatment for 48 h, were collected, which were designated to OC exo and SK OC exo, respectively. **A**, morphology of isolated OC exo or SK OC exo determined under TEM; **B**, particle size distribution and concentration of isolated OC exo or SK OC exo examined by NTA; **C**, protein expression of exo marker proteins CD9, CD63 and CD81 and endoplasmic reticulum marker Calnexin in the isolated OC exo or SK OC exo determined by WB analysis; **D**, successful uptake of DiO-labeled exo by THP-1 cells observed under the fluorescence microscope. THP-1 cells were stimulated with 150 nM PMA for 24 h to differentiate into M0 macrophages, followed by treatment with PBS, OC exo or SK OC exo. **E**-**F**, percentage of CD163- (**E**) and CD206-(**F**) positive THP-1 cells examined by flow cytometry; **G**, production of IL-10 in the culture supernatant of THP-1 cells examined using ELISA; **H**, mRNA expression of CD163 and CD206 in THP-1 cells determined by RT-qPCR. Three biological replicates were performed. Differences were analyzed by the one-way or two-way ANOVA. *p < 0.05

As introduced earlier, tumor cell-derived exo can induce immunosuppression and further favor tumor progression. Here, we further investigated whether SK treatment influences the OC cell-derived exo. To begin with, exo from SKOV3 and A2780 cells, either with or without 5 µM SK treatment for 48 h, were collected, which were designated to OC exo and SK OC exo, respectively. Under the TEM, the isolated particles appeared as disc-shaped membrane vesicles (Fig. 2A). In addition, according to NTA, the particle size was mainly distributed to around 100 nm, and the SK treatment significantly reduced the particle secretion by OC cells (Fig. 2B). WB analysis verified that these collected particles exhibited positive expression of exo-specific markers CD9, CD63 and CD81 while having no expression of the endoplasmic reticulum marker Calnexin (Fig. 2C). We treated the PMA-stimulated THP-1 cells (all THP-1 cells mentioned hereafter are PMA-stimulated ones) with the exo. The DiO-labeled exo were successfully taken up by the THP1 cells (Fig. 2D). Importantly, the OC exo treatment significantly increased the M2 polarization markers CD163 and D206 in the THP-1 cells, while this increase was substantially decreased when the OC cells had been treated with SK (Fig. 2E-F). ELISA results showed that the production of IL-10 in the THP-1 cells was increased by the OC exo treatment, and the SK treatment in the OC cells reduced this increase (Fig. 2G). RT-qPCR also verified that the CD163 and CD206 expression in the THP-1 cells was elevated by OC exo as well. However, compared to the OC exo, the SK OC exo reduced the CD163 and CD206 expression (Fig. 2H). This evidence suggests that the SK treatment can reduce the secretion of OC cell-derived exo and reduce the promoting effect of these exo on M2 polarization of macrophages.

### SK treatment reduces GAL protein level in OC exo

**Fig. 3:**
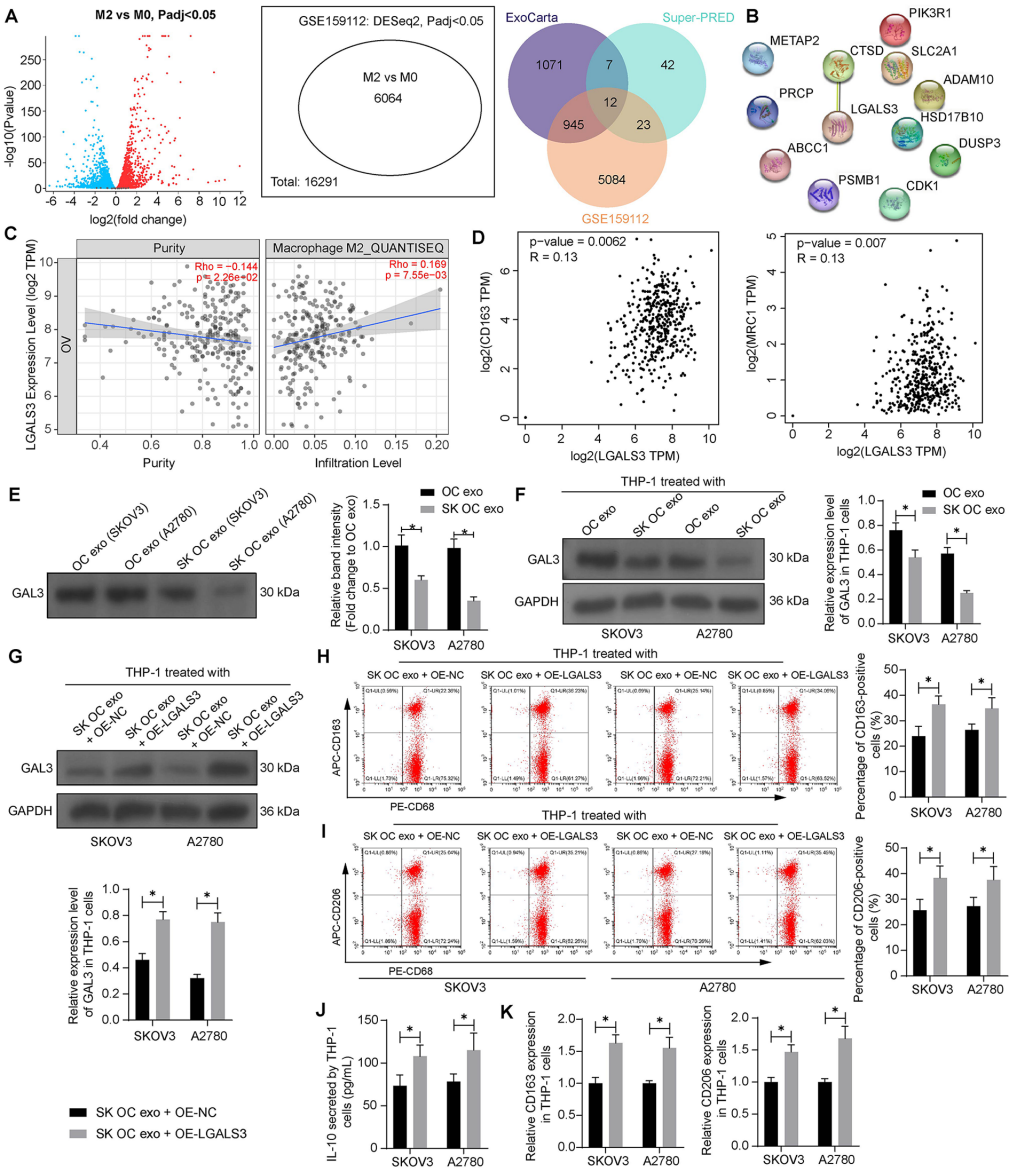
SK treatment reduces GAL3 protein level in OC exo. **A**, intersections of molecular targets of SK predicted from Super-PRED system, proteins enriched in OC cell (OVCAR-3 and IGROV1)-derived exo obtained from the ExoCarta system, and differentially expressed genes between M0 and M2 macrophages obtained by analyzing the GEO GSE159112 dataset; **B**, protein-protein interaction between the 12 intersections analyzed using the STRING system; **C**, a positive correlation between LGALS3 and M2 macrophage polarization in OC predicted using the TIMER system; **D**, correlations between LGALS3 expression and CD163 and CD206 expression in OC predicted using the GEPIA system; **E**, GAL3 protein level in OC exo and SK OC exo determined by WB analysis (the band intensity of GAL3 in OC exo was used for normalization); **F**, GAL3 protein level in THP-1 cells after OC exo or SK OC exo treatment examined by WB analysis. THP-1 cells treated with SK OC exo were further transfected with OE-LGALS3 or OE-NC. **G**, GAL3 protein level in THP-1 cells determined by WB analysis; **H**-**I**, percentage of CD163 (**H**)- and CD206 (**I**)-positive THP-1 cells determined by flow cytometry; **J**, production of IL-10 in the culture supernatant of THP-1 cells examined using ELISA; **K**, mRNA expression of CD163 and CD206 in THP-1 cells determined using RT-qPCR. Three biological replicates were performed. Differences were analyzed by the two-way ANOVA. *p < 0.05

To identify the molecular target of SK, we predicted 84 possible chemical targets of it using the Super-PRED system (https://prediction.charite.de/index.php?site=chemdoodle_search_target). In addition, a list of 2035 proteins enriched in OC (OVCAR-3 and IGROV1 cells)-derived exo were obtained from the ExoCarta system (http://www.ncell.com.cn/index.php?case=archive&act=show&aid=24). The two sets of proteins were cross-referenced with differentially expressed genes (*n* = 6064) between M0- and M2-phenotype THP-1-derived macrophages obtained using the GEO GSE159112 dataset (https://www.ncbi.nlm.nih.gov/geo/query/acc.cgi?acc=GSE159112), and a total of 12 intersections were obtained (Fig. 3A). A protein-protein interaction network based on the 12 factors was generated using the STRING system (https://string-db.org/), and the one and only interaction was found between LGALS3 and CTSD (Fig. 3B). LGALS3/GAL3 attracted our attention as it has been reportedly correlated with M2 polarization of macrophages [20]. Data from the TIMER (https://cistrome.shinyapps.io/timer/) system suggests a significant positive correlation between LGALS3 (galectin 3 gene) and M2 macrophage infiltration in OC (Fig. 3C). In addition, the GEPIA system (http://gepia.cancer-pku.cn/index.html) suggests positive correlations of LGALS3 with CD163 and CD206 in OC (Fig. 3D). Importantly, WB analysis showed that the protein level of GAL3 was significantly reduced in the SK OC exo compared to the OC exo (Fig. 3E). Correspondingly, the GAL3 protein level in THP-1 cells was reduced after SK OC exo treatment compared to the OC exo treatment (Fig. 3F). The SK OC exo-treated THP-1 cells were further transfected with OE-LGALS3, which successfully elevated the GAL3 expression (Fig. 3G). Importantly, the LGALS3 overexpression significantly increased the percentage of M2-type macrophages, as manifested by increased CD163 and CD206 expression in the THP-1 cells (Fig. 3H-I). Likewise, the IL-10 production was increased under the condition of LGALS3 overexpression as well (Fig. 3J). The CD163 and CD206 mRNA expression in the THP-1 cells was increased as well following LGALS3 overexpression (Fig. 3K).

### GAL promotes nuclear translocation of β-catenin to induce M2 polarization of macrophages

**Fig. 4:**
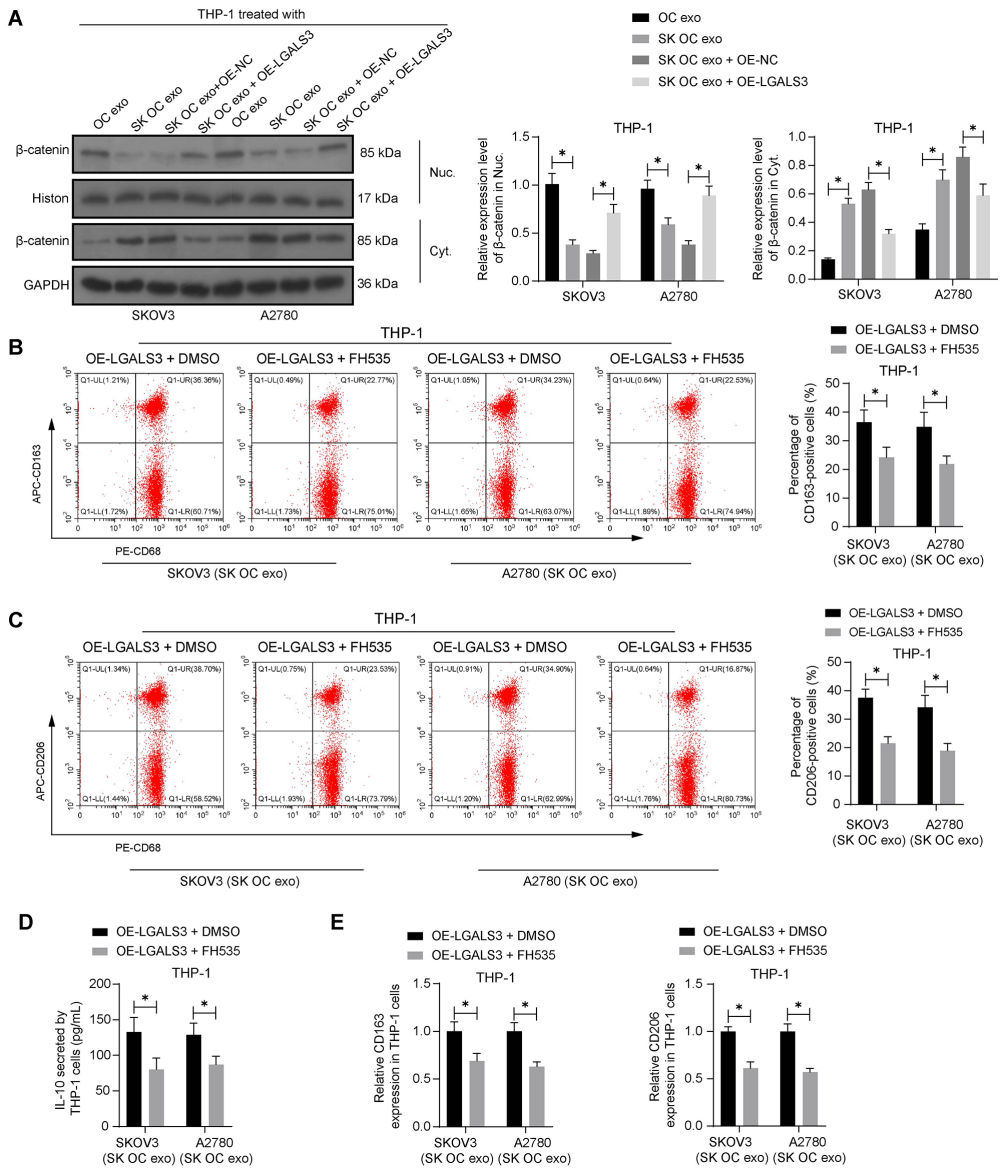
GAL3 promotes nuclear translocation of β-catenin to induce M2 polarization of macrophages. **A**, nuclear and cytoplastic β-catenin levels in THP-1 cells after OC exo or SK OC exo treatment determined by WB analysis. THP-1 cells treated with SK OC exo and transfected with OE-LGALS3 were further treated with the β-catenin inhibitor FH535 or DMSO. **B**-**C**, percentage of CD163 (**B**)- and CD206 (**C**)-positive THP-1 cells examined by flow cytometry; **D**, production of IL-10 in the culture supernatant of THP-1 cells examined using ELISA; **E**, mRNA expression of CD163 and CD206 in THP-1 cells determined by RT-qPCR. Three biological replicates were performed. Differences were analyzed by the two-way ANOVA. *p < 0.05

Interestingly, GAL3 has been associated with nuclear translocation of β-catenin [21], and the β-catenin signal transduction is closely correlated with M2 polarization of macrophages [22]. Therefore, we surmised that SK might also suppress the GAL3/β-catenin interaction to modulate macrophage phenotype. Importantly, WB analysis showed that the nuclear protein level of β-catenin was substantially decreased in THP-1 cells after SK OC exo treatment compared to the OC exo treatment; however, nuclear β-catenin level was restored by LGALS3 overexpression (Fig. 4A). Correspondingly, the cytoplastic distribution of β-catenin was increased by SK OC exo but reduced by OE-LGALS3 (Fig. 4A).

In the presence of SK OC exo and OE-LGALS3 treatments, THP-1 cells were further treated with the β-catenin inhibitor FH535. Compared to DMSO, the FH535 significantly reduced the percentage of M2-polarized macrophages (Fig. 4B-C), along with a decrease in the production of IL-10 (Fig. 4D). The mRNA expression of CD163 and CD206 in the cells was decreased by the FH535 as well (Fig. 4E).

### SK treatment reduces tumorigenesis of SKOV3 cells in nude mice and reduces M2 macrophage infiltration

**Fig. 5:**
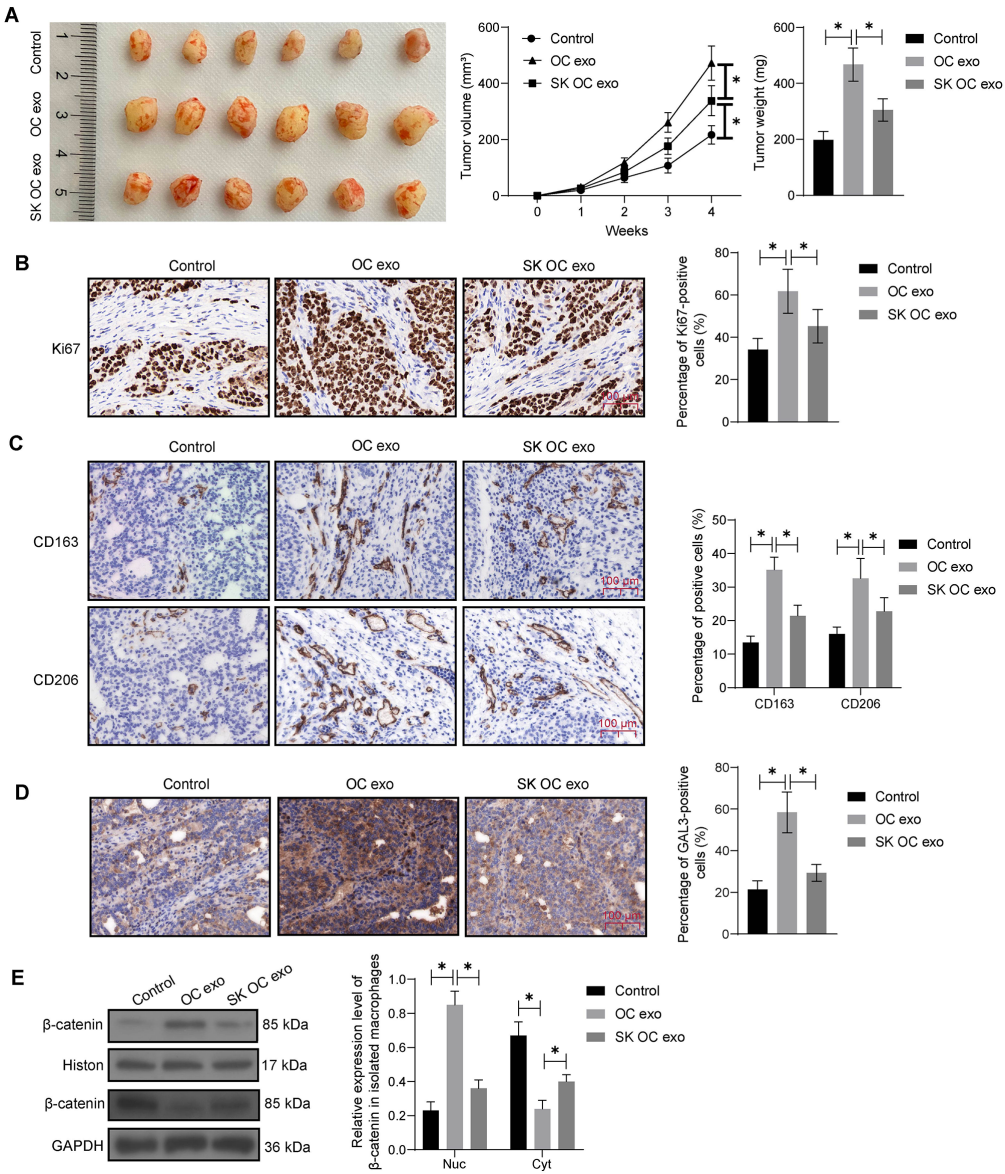
SK treatment reduces tumorigenesis of SKOV3 cells in nude mice and reduces M2 macrophage infiltration. SKOV3 cells were subcutaneously injected into CB-17 SCID mice to generate xenograft tumors, followed by OC exo or SK OC exo treatment. **A**, growth rate within 4 weeks and weight at week 4 of xenograft tumors in mice; **B**, Ki67 expression in the xenograft tumor tissues examined by IHC; **C**, expression of CD163 and CD206 in the xenograft tumor tissues examined by IHC; **D**, GAL3 expression in the xenograft tumor tissues examined by IHC; **E**, nuclear and cytoplastic β-catenin levels in macrophages isolated from xenograft tumors determined by WB analysis. In each group, *n* = 6. Differences were compared by the one-way or two-way ANOVA. *p < 0.05

SKOV3 cells were injected into mice to establish subcutaneous xenograft tumor models, and the mice were left untreated, or treated with OC exo or SK OC exo. Compared to the untreated mice, the mice treated with OC exo had increased tumor growth rate and tumor volume after four weeks. However, compared to the OC exo group, the xenograft tumorigenesis was retarded in the SK OC exo group (Fig. 5A). IHC showed that the expression of the tumor proliferation marker Ki67, the M2 polarization markers CD163 and CD206, and GAL3 in the xenograft tumor tissues was significantly increased by the OC exo treatment, but this increase was decreased in the SK OC exo group as well (Fig. 5B-D), mirroring the observations in vitro. In addition, the macrophages from xenograft tumors were isolated, and the nuclear β-catenin level was found to be increased by OC exo, but this increase was less pronounced in the SK OC exo group (Fig. 5E).

## Discussion

Although the tumor-suppressive effect of SK has been well established in human cancers [10], including OC [11], its functional mechanisms have not been fully elucidated. In this study, the authors report that SK suppresses malignant phenotype of OC cells and reduces M2-shifted macrophages, which entails the inhibition of exo production and exosomal protein GAL3.

SK has anti-oxidative, anti-inflammatory, and anti-cancer properties, and it possesses anti-cancer effects primarily by inducing cell cycle arrest, apoptosis, autophagy, and necroptosis while inhibiting cell proliferation and dissemination [23]. In our investigation, we observed notable cytotoxicity of SK against OC cell lines, evidenced by IC50 values of 9.33 µM for SKOV3 cells and 10.73 µM for A2780 cells. These findings align partially with those reported by Hao et al. [24] and Shilnikova et al. [12]. To minimize potential toxic effects while still observing drug efficacy, doses below the IC50 value have been deemed reasonable [25, 26]. Consequently, we employed a fixed dose of SK at 5 µM for 48 h to investigate its impact on OC cells. This treatment regimen significantly inhibited proliferation, migration, and invasion, as well as conferred a reduction in apoptosis resistance and tumorigenic potential of OC cells. Notably, similar trends have been observed in OC cells [11, 24] and other cancer cell types, such as prostate cancer [27], upon administration of SK at this dosage. Moreover, these studies and others have demonstrated that the cytotoxicity of SK against cancer cells can be dose- and time-dependent [28, 29]. However, this point was not further investigated in this study and should be acknowledged as a shortcoming.

To date, several molecular mechanisms have been implicated in its anti-tumor events. SK has been recognized as a potent and selective inhibitor of pyruvate kinase isozyme M2 (PKM2), which is highly expressed in many human tumors and participates in aerobic glycolysis, and cell proliferation [30, 31]. SK has been found to inactivate the IL-6/signal transducer and activator of transcription 3 signaling and decrease A disintegrin and metalloproteinase 17 expression to suppress growth of colon cancer cells [28]. Wang and colleagues demonstrated that SK suppressed cell cycle progression and cell growth by upregulating P21, a potent inhibitor of cyclin-dependent kinases that induces cell cycle arrest in G1 or G2/M phase [29]. In OC, SK has been found to induce PKM2 silencing, therefore possessing a synergistic effect with the chemo drug Olaparib to inhibit tumor cell growth and migration [32]. It also presented a synergistic tumor inhibiting effect with paclitaxel on OC cells in a P-glycoprotein-independent manner [33]. Additionally, the tumor suppressive effect of SK in OC entails the inhibition of G protein-coupled estrogen receptor signaling [11].

Notably, SK has been reported to reduce tumor-derived exo to inhibit proliferation and progression of breast cancer cells [13]. Not come singly but in pairs, SK has been found to reduce exo secretion and the exosomal PKM2 level, therefore suppressing glycolytic flux and chemoresistance in non-small cell lung cancer [34]. Ramkishore and colleagues also found that SK reduced the preadipocyte-derived exosomal secretion of growth factors and cytokines that are directly correlated with angiogenesis, tumor growth, and invasion [35]. These facts triggered our concerns to investigate the effect of SK on exo secretion in OC. Importantly, the SK treatment decreased the secretion of exo by OC cells. Exo are key factors involved in intracellular communication and can reprogram the TME, while altered production or composition of tumor-derived exo can alter the fate of nearby cells and affect the immune response and the hematopoietic system [36]. In general, they deliver immunosuppressive cytokines into the TME and induce M2 polarization of macrophages, which in turn favors tumor growth and dissemination [37, 38]. This situation has been applied in OC as well. A previous study by Chen et al. suggests the exo secreted by SKOV3 cells were taken up by macrophages, and the exo co-culture led to increased CD163 and CD206 levels in M0 macrophages, especially under a hypoxic condition [39]. Notably, by using PMA-stimulated THP-1 cells, we found that the SK treatment significantly reduced the promoting effects of OC cell-derived exo on M2 skewing of macrophages, as manifested by decreased levels of CD163, CD206, and IL-10. The SK OC exo also led to reduced tumorigenesis and M2 macrophage population compared to OC exo in a xenograft mouse model. Intriguingly, the SK derivatives have been demonstrated to protect immune organs while promoting immune response in tumor-bearing mice [40]. In addition, by suppressing PKM2, SK has been found to reduce the programmed cell death-ligand 1 level on macrophages [41]. Here, this study demonstrates that SK possesses an immune regulatory role in OC by decreasing M2 skewing of macrophages through decreasing exo secretion.

When exploring the specific molecule targeted by SK in the M2 suppressing events, we performed comprehensive bioinformatics analysis, with LGALS3 identified as a promising target. LGALS3 encodes the GAL3 protein, which is a carbohydrate-binding protein with an affinity for N-acetyllactosamine residues. This protein is unique because it is associated with tumor progression and metastasis by regulating various processes, including proliferation, survival, dynamic cellular transformation, migration, invasion, and stemness [42, 43]. Specifically, GAL3 has also been correlated with M2 polarization of macrophages [20, 44, 45]. In addition, GAL3 has been reported to increase nuclear translocation and activation of β-catenin by promoting glycogen synthase kinase-3beta phosphorylation and reducing its activity [21], and GAL3-mediated β-catenin has been correlated with metastasis of hepatocellular carcinoma [46]. The exosomal GAL3 level has also been correlated with β-catenin level [47]. The aberrant activation of β-catenin signaling is closely correlated with various human diseases, including cancer, and it participates in a wide array of tumorigenic events from onset to development [48]. This includes its promoting role in M2 polarization of macrophages [49, 50]. Interestingly, SK has also been found to downregulate β-catenin expression, therefore enhancing the chemo sensitivity of oral cancer cells [51]. Importantly, in this study, we found that the SK treatment in OC cells decreased the exosomal protein level of GAL3, which led to a decrease in GAL3 level in the recipient macrophages, along with decreased nuclear translocation of β-catenin. The facts that the M2 polarization of macrophages was restored by LGALS3 overexpression but reduced by the β-catenin validated that the GAL3/β-catenin interaction is, at least partly, entailed in the events mediated by SK.

## Conclusion

In conclusion, this study verifies that SK does have a treatment effect on OC cells. Most of all, this research provides novel evidence that SK can reduce M2 polarization of macrophages by decreasing the secretion of OC cell-derived exo, or by reducing the exosomal level of GAL3 and the consequent β-catenin activation. However, due to constraints in time and finances, we did not delve into the time- or dose-dependent effects of SK on OC cells, OC exo, and the phenotypic shift of macrophages, representing a significant limitation of this study that warrants future investigation. Nevertheless, the insights gleaned from this research offer valuable contributions to understanding the pharmacological mechanism of SK in cancer treatment.

## Materials and methods

### Cell sources and culture

OC cell lines SKOV3 (CL-0215) and A2780 (CL-0013), and a human monocytic leukemia cell line THP-1 (CL-0233), were acquired from Procell Life Science & Technology Co., Ltd. (Wuhan, Hubei, China). OC cells were cultured in DMEM (Thermo Fisher Scientific, Rockford, IL, USA) supplemented with 10% fetal bovine saline (FBS) and 1% antibiotics in a humidified environment at 37° with 5% CO_2_. Exponentially growing cells were used for subsequent experiments. THP-1 cells were incubated with 150 nM phorbol 12-myristate 13-acetate (PMA, ab120297, Abcam Inc., Cambridge, MA, USA) for 24 h and then cultured in complete RPMI-1640 for 24 h to differentiate into M0 macrophages. For SK treatment, every 1 × 10^4^ OC cells were treated with 5 µM of SK (HY-N0822, MedChemExpress, Monmouth Junction, NJ, USA) dissolved in dimethyl sulfoxide (DMSO) for 48 h. For exo (see preparation details later) treatment, every 1 × 10^4^ THP-1 cells were treated with 50 µg exo for 72 h. Cells without SK or exo treatment were set to controls. For β-catenin inhibition, THP-1 cells were treated with 10 µM of β-catenin/Tcf inhibitor FH535 (219330, Sigma-Aldrich, Merck KGaA, Darfmstadt, Germany) for 12 h, and those treated with an equal volume of DMSO solution were set to controls. All procedures were conducted in a sterile condition.

### Cell transfection

Approximately 1 × 10^5^ THP-1 cells treated with SK OC exo (see details later) were seeded in plates containing 500 µL of culture medium. The overexpression plasmid of galectin 3 (OE-LGALS3) or the negative control (OE-NC) was transfected into cells using the Lipofectamine™ LTX reagent with PLUS™ reagent (15,338,100, Thermo Fisher Scientific). For each transfection sample, the complex was prepared as follows: plasmid DNA was diluted in 100 µL of Opti-MEM® I reduced-serum medium at an optimized dose and thoroughly mixed. The PLUS™ reagent was gently mixed and added to the diluted DNA at the optimized volume, followed by gentle mixing and incubation at room temperature for 5 min. Lipofectamine® LTX was gently mixed and then added to the diluted DNA, followed by mixing and an incubation at room temperature for 30 min. The DNA-LPS complex is stable for 6 h at room temperature. Approximately 100 µL of DNA-liposome complex was added dropwise to the wells containing cells. The plate was gently shaken back and forth for mixing. The cells were then incubation at 37 °C in a CO_2_ incubator before further analyses.

### Cell counting kit-8 (CCK-8) method

The cytotoxicity of SK to OC cells was measured using the CCK-8 kit (V13154, Thermo Fisher Scientific). Briefly, the SKOV3 or A2780 cells were seeded in 12-well plates and at 1 × 10^4^ cells per well. After 12 h, each well was added with ascending series doses (1 µM, 2 µM, 4 µM, 8 µM, 16 µM, 32 µM, 64 µM, 128 µM, and 256 µM) of SK for 48 h [24], followed by the addition of 10 µL CCK-8 reagent for another 2 h of incubation at 37 °C. The optical density (OD) at 450 nm was read and the 50% inhibitory concentration (IC50). For the rest behavior tests, the SKOV3 or A2780 cells were treated with a fixed dose (5 µM) of SK instead.

### Colony formation assay

The treated SKOV3 or A2780 cells were seeded into six-well plates and cultured at 37 °C with 5% CO_2_ for two weeks. Later, the cells were fixed with 4% paraformaldehyde for 15 min and stained with crystal violet (R40073, Thermo Fisher Scientific) for 10 min. The number of cell colonies was determined under a microscope.

### Wound healing assay

The treated SKOV3 or A2780 cells (200 µL suspension containing 1 × 10^4^ cells) were seeded in culture dishes. When a cell monolayer was formed, a scratch was produced using a pipette tip. The cell debris was then washed away, and the wound width at 0 h was photographed and recorded. After 24 h of cell incubation in serum-free medium, the wound width was determined again to calculate the 24-h migration rate of cells.

### Transwell invasion assay

Approximately 1 × 10^4^ treated SKOV3 or A2780 cells were suspended in 200 µL serum-free medium and added to 24-well Transwell apical chambers, which were pre-coated with Matrigel (Sigma-Aldrich). The basolateral chambers were added with FBS-containing medium as attractant. After 24 h of incubation, the cells invaded the lower membranes were fixed with 4% paraformaldehyde and stained with 1% crystal violet for 15 min, and the number of invading cells was calculated under a microscope.

### Isolation of OC cell-derived exo

OC cells were subjected to continuous low- or middle-speed centrifugation to discard dead cells or cell debris. Subsequently, 5 mL of total exo isolation reagent (4478359, Thermo Fisher Scientific) was added to 10 mL of cultured cells for an overnight incubation at 2℃ to 8℃. The exo were isolated after a 60-min ultra-centrifugation at 100,000 *g* and then resuspended in phosphate-buffered saline (PBS) for further use. For authentication, 10 µg of exosomal protein was collected for western blot (WB) analysis, focusing on the exo-specific positive markers CD9, CD63, and CD81 and negative marker Calnexin.

### Transmission electron microscope (TEM) examination

Morphology of the isolated particles was observed under a TEM. In brief, 10 µL of particle solution was first loaded onto an electron microscopy grid and air-dried at 37 °C for 30 min to precipitate the exosomes. Subsequently, fixation was carried out using 1% glutaraldehyde (Sigma, 1.12179) for 5 min. Following this, the particles were contrasted for 10 min with a mixture of uranyl acetate and methyl cellulose. Excess liquid was removed with filter paper and dried. Finally, the particles were observed under an 80 kV working voltage using a JEM 1010 transmission electron microscope (JEOL USA, Inc., Peabody, MA).

### Nanoparticle tracking analysis (NTA) analysis

The extracted particles were diluted with PBS to a final volume of 1 mL. Using a sterile syringe, the particles were loaded into the sample chamber for real-time monitoring of NTA acquisition. NanoSight NTA1 software (Malvern Instruments Ltd, Worcestershire, UK) was employed to track and capture videos of all particles within the field of view. All measurements were conducted at temperatures ranging from 24 °C to 26 °C, with an injection speed of 22 µL/s and using a 488 nm laser. In the information provided by the software, the average size, mode (the most representative size population), and particle concentration (particles/mL) were analyzed.

### Terminal deoxynucleotidyl transferase (TdT)-mediated dUTP nick end labeling (TUNEL)

According to the instruction manual of the TUNEL assay kit (C1089, Beyotime), the OC cells were seeded on glass slides at 1 × 10^4^ cells/mL and cultured for 24 h, followed by SK treatment. Later, the cells were fixed for 30 min and penetrated with 0.2% Triton X-100 solution for 5 min. Subsequently, each well was added with 50 µL of prepared TUNEL reagent for 60 min of incubation in the dark at 37℃. The positive labeling was observed under the Nikon eclipse 80i fluorescence microscope (Nikon Instruments Inc., Tokyo, Japan) at an excitation wavelength of 550 nm and an emission wavelength of 570 nm.

### WB analysis

The isolated exo or THP-1 cells were lysed in cold lysis buffer, and the lysates were centrifuged at 2,000 *g* to collect total protein. After concentration measurement using the bicinchoninic acid (23229, Thermo Fisher Scientific), an equal amount of protein sample was separated by SDS-PAGE and transferred onto polyvinylidene fluoride membranes. After blocking using 5% normal goat serum, the membranes were probed with the antibodies against CD9 (1:1000, ab236630, Abcam), CD63 (1:1000, ab134045, Abcam), CD81 (1:1000, ab79559, Abcam), Calnexin (1:1000, PA5-34665, Thermo Fisher Scientific), Histone H3 (1:1000, ab1791, Abcam), GAL3 (1:2000, ab76245, Abcam), glyceraldehyde-3-phosphate dehydrogenase (GAPDH, 1:5000, ab8245, Abcam), and β-catenin (1:5000, ab32572, Abcam) at 4℃ overnight, followed by incubation with the rabbit anti-mouse immunoglobulin G (IgG, 1:1000, #58802S, Cell Signaling Technology [CST], Beverly, MA, USA) or goat anti-rabbit IgG (1:2000, 7074, CST) at room temperature for 1 h. The protein bands were developed using enhanced chemiluminescence reagent. The grayscale values (band intensity) was measured using Image J to evaluate the relative expression of target proteins, with GAPDH used as the loading control for normalization. Specifically, for the detection of β-catenin, its protein level in nucleus and cytoplasm was determined, respectively, by separating the nuclear and cytoplasmic components of THP-1 cells or of macrophages isolated from xenograft tissues (see animal models later). In this case, Histone H3 was used as the loading control for nuclear protein while GAPDH for cytoplasmic protein.

### Exo uptake assay

The exo (50 µg) were incubated with the lipophilic tracer DiO solution (V22886, Thermo Fisher Scientific) at 37 °C for 20 min. The DiO-labeled exo were then added to the culture medium of recipient cells (THP-1) for 3 h of incubation at 37 °C. Later, the THP-1 cells were fixed with 4% paraformaldehyde at room temperature for 10 min and permeabilized with 0.1% Triton X-100 for 5 min. The cells were then stained with wheat germ agglutinin (WGA, W849, Thermo Fisher Scientific) conjugated to a fluorescent dye at room temperature for 30 min. The slides were mounted with Prolong™ Live Antifade Reagent (P36975, Thermo Fisher Scientific) and covered with a coverslip for observation under the Nikon eclipse 80i fluorescence microscope (DiO: excitation/emission = 480/510 nm; WGA: excitation/emission = 555/580 nm).

### Flow cytometry

The THP-1 cells were cultured in DMEM containing 10% FBS in 24-well plates (1 × 10^5^ cells per well). The untreated THP-1 cells (Normal), those treated with exo derived from OC cells (OC exo) or derived from SK-treated OC cells (SK OC exo) for 4 h, those further transfected with OE-LGALS3 or OE-NC, or those further treated with β-catenin or DMSO were resuspended in binding buffer. The cells were incubated with antibodies of phycoerythrin-labeled CD68 (1:20, 12-0689-41, Thermo Fisher Scientific), allophycocyanin-labeled CD163 (1:10, ab134416, Abcam), and allophycocyanin-labeled CD206 (1:100, 17-2069-42, Thermo Fisher Scientific) for 30 min. The cells were then resuspended in 500 µL of binding buffer and analyzed using flow cytometry (BD, FACSCalibur, USA) within 1 h to analyze polarization of macrophages.

### Enzyme-linked immunosorbent assay (ELISA)

The interleukin (IL)-10 concentration in the culture supernatant of THP-1 cells with or without SK OC exo treatment was determined using a human IL-10 ELISA kit (88-7106-88, Thermo Fisher Scientific). All procedures were conducted in strict accordance with the manufacturer’s instructions. The OD value at 450 nm was read using a microplate reader.

### Reverse transcription quantitative polymerase chain reaction (RT-qPCR)

Total RNA from THP-1 cells was extracted using the TRIzol reagent, and the cDNA was synthesized using the PrimeScript™ RT Master Mix (RR036Q, Takara). Subsequently, real-time qPCR was performed using the PowerUp™ SYBR™ Green Premix (A25742, Thermo Fisher Scientific) and a real-time qPCR examination system (Bio-Rad Laboratories, Hercules, CA, USA). Relative gene expression, with β-actin served as the reference gene, was evaluated using the 2^−ΔΔCt^ method. The primer sequence information is listed below: CD163 (F): 5ʹ-CCAGAAGGAACTTGTAGCCACAG-3ʹ, (R) 5ʹ-CAGGCACCAAGCGTTTTGAGCT-3ʹ; CD206 (F): 5ʹ-AGCCAACACCAGCTCCTCAAGA-3ʹ, (R) 5ʹ-CAAAACGCTCGCGCATTGTCCA-3ʹ, β-actin (F) 5ʹ-CACCATTGGCAATGAGCGGTTC-3ʹ, (R) 5ʹ-AGGTCTTTGCGGATGTCCACGT-3ʹ.

### Xenograft tumors in nude mice

CB-17 SCID mice (4–6 weeks old) were acquired from Vital River Laboratory Animal Technology Co., Ltd. (Beijing, China) and used in guidelines approved by the Animal Ethics Committee of Wuxi Maternity and Child Health Care Hospital. The mice were housed in a specific pathogen-free facility with access to abundant food and water and regular bedding changes. Each cage housed no more than 5 mice. After one week of acclimation, 18 mice were divided into three groups of six mice each: Control group, OC exo group, and SK OC exo group. To induce xenograft tumors, each mouse was subcutaneously injected with 0.2 mL of single cell suspension of SKOV3 cells (1 × 10^6^ cells /mL). Mice in the Control group left untreated, while those in the OC exo and SK OC exo groups were treated with 20 mg/kg OC exo or SK OC exo, respectively, once a week by intravenous injection. Tumor volumes were measured at weeks 0, 1, 2, 3, and 4. The tumor length (L) and width (W) were measured using calipers, and tumor volume (V) was calculated using the formula: L × (W)^2^/2. At the end of week 4, the mice were euthanized by intraperitoneal injection of an overdose of nembutal (150 mg/kg) to collect and weight the tumors.

### Immunohistochemistry (IHC)

The harvested xenograft tumor tissues were paraffined and sectioned, and the sections were de-paraffined and rehydrated. After antigen retrieval using a citrate buffer solution in a microwave and endogenous peroxidase blockage using a 3% hydrogen peroxide, the slides were further blocked with 5% goat serum at room temperature for 2 h. Subsequently, the sections were incubated with the antibodies of Ki67 (1:1000, ab15580, Abcam), GAL3 (1:2000, ab76245, Abcam), CD163 (1:200, ab182422, Abcam), and CD206 (1:500, #24595, CST) overnight at 4 °C, followed by incubation with rabbit anti-mouse IgG (1:1000, #58802S, CST) or goat anti-rabbit IgG (1:2000, 7074, CST) at room temperature for 1 h. After color development using DAB, the sections were counter-stained with hematoxylin, dehydrated with ethanol, cleared with xylene, and mounted with a neutral mountant under a light microscope. The percentage of positive cells under the view, stained in brownish, was then calculated.

### Statistical analysis

All data were analyzed using Prism 8.0.2 software (GraphPad, La Jolla, CA, USA). All data were collected from no less than three independent experiments and are exhibited as the mean ± standard deviation. Differences were analyzed using the one- or two-way analysis of variance (ANOVA) followed by Tukey’s post-hoc check. Statistical significance was defined by *p* < 0.05.

## Electronic supplementary material

Below is the link to the electronic supplementary material.

Supplementary Material 1

Supplementary Material 2

## Data Availability

The analyzed data generated during this study are available from the corresponding author on reasonable requests.

## Abbreviations

ANOVA: analysis of variance
CCK-8: cell counting kit-8
ELISA: enzyme-linked immunosorbent assay
exo: exosomes
DMSO: dimethyl sulfoxide
FBS: fetal bovine saline
GAPDH: glyceraldehyde-3-phosphate dehydrogenase
IHC: immunohistochemistry
IL-10: interleukin-10
LGALS3: galectin 3
NTA: nanoparticle tracking analysis
OC: ovarian cancer
OC exo: exosomes isolated from untreated ovarian cancer cells
OD: optical density
PBS: phosphate-buffered saline
PMA: phorbol 12-myristate 13-acetate
RT-qPCR: reverse transcription quantitative polymerase chain reaction
SK: Shikonin
SK OC exo: exosomes isolated from Shikonin-treated ovarian cancer cells
TEM: transmission electron microscope
TME: tumor microenvironment
TUNEL: Terminal deoxynucleotidyl transferase (TdT)-mediated dUTP nick end labeling
WB analysis: western blot analysis
