# Correction: Shikonin reduces M2 macrophage population in ovarian cancer by repressing exosome production and the exosomal galectin 3-mediated β-catenin activation

**DOI:** 10.1186/s13048-025-01652-z

**Published:** 2025-03-27

**Authors:** Min Wang, Yangyan Sun, Rui Gu, Yan Tang, Guorong Han, Shaojie Zhao

**Affiliations:** 1 Department of Gynaecology and Obstetrics, The Second Hospital of Nanjing, Nanjing University of Chinese Medicine, No.1, Zhongfu Road, Nanjing, Jiangsu 210003 P.R. China; 2 Department of Gynaecology, Wuxi Maternity and Child Health Care Hospital, Affiliated Women’s Hospital of Jiangnan University, No. 48, Huaishu Lane, Liangxi District, Wuxi, Jiangsu 214000 P.R. China; 3 Department of Gynecology, Jiangyin People’s Hospital, Wuxi, Jiangsu 214400 P.R. China

**Correction:** ***J Ovarian Res*** **17, 101 (2024)**

10.1186/s13048-024-01430-3

Following publication of the original article [1], the authors reported that the second affiliation of author Min Wang is not presented in the published article. Below is the correct author group section.

**Incorrect:**

Min Wang^1†^, Yangyan Sun^2†^, Rui Gu^1^, Yan Tang^1^, Guorong Han^3*^ and Shaojie Zhao^1*^

^1^Department of Gynaecology, Wuxi Maternity and Child Health Care Hospital, Affiliated Women’s Hospital of Jiangnan University, No. 48, Huaishu Lane, Liangxi District, Wuxi, Jiangsu 214,000, P.R. China.

^2^Department of Gynecology, Jiangyin People’s Hospital, Wuxi, Jiangsu 214,400, P.R. China.

^3^Department of Gynaecology and Obstetrics, The Second Hospital of Nanjing, Nanjing University of Chinese Medicine, No.1, Zhongfu Road, Nanjing, Jiangsu 210003, P.R. China.

**Correct:**

Min Wang^1,2†^, Yangyan Sun^3†^, Rui Gu^2^, Yan Tang^2^, Guorong Han^1*^ and Shaojie Zhao^2*^

^1^Department of Gynaecology and Obstetrics, The Second Hospital of Nanjing, Nanjing University of Chinese Medicine, No.1, Zhongfu Road, Nanjing, Jiangsu 210003, P.R. China.

^2^Department of Gynaecology, Wuxi Maternity and Child Health Care Hospital, Affiliated Women’s Hospital of Jiangnan University, No. 48, Huaishu Lane, Liangxi District, Wuxi, Jiangsu 214,000, P.R. China.

^3^Department of Gynecology, Jiangyin People’s Hospital, Wuxi, Jiangsu 214,400, P.R. China.

The original article [1] has been corrected.
