# Supplementary material for: Shikonin reduces M2 macrophage population in ovarian cancer by repressing exosome production and the exosomal galectin 3-mediated β-catenin activation
Source: J Ovarian Res. 2024 May 14;17:101. doi: 10.1186/s13048-024-01430-3 (PMC11092256; doi:10.1186/s13048-024-01430-3)

**Supplementary Fig S1A** A whole plate of colonies formed by SKOV3 and A2780 cells after treatment with 5 µM of SK for 48 h.


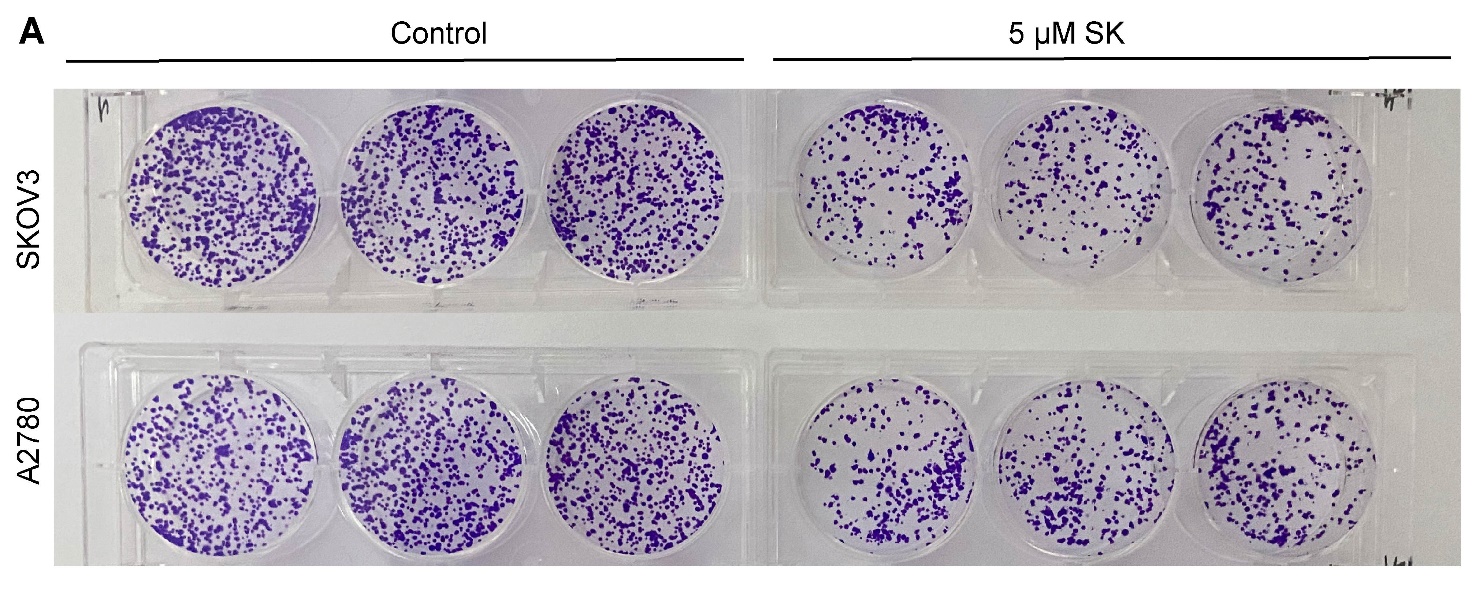

Supplement: Supplementary file 1 — Supplementary Material 1 [file 13048_2024_1430_MOESM1_ESM.docx]
