# Supplementary material for: Shikonin reduces M2 macrophage population in ovarian cancer by repressing exosome production and the exosomal galectin 3-mediated β-catenin activation
Source: J Ovarian Res. 2024 May 14;17:101. doi: 10.1186/s13048-024-01430-3 (PMC11092256; doi:10.1186/s13048-024-01430-3)

The raw data of western blot

Fig 2C

CD9

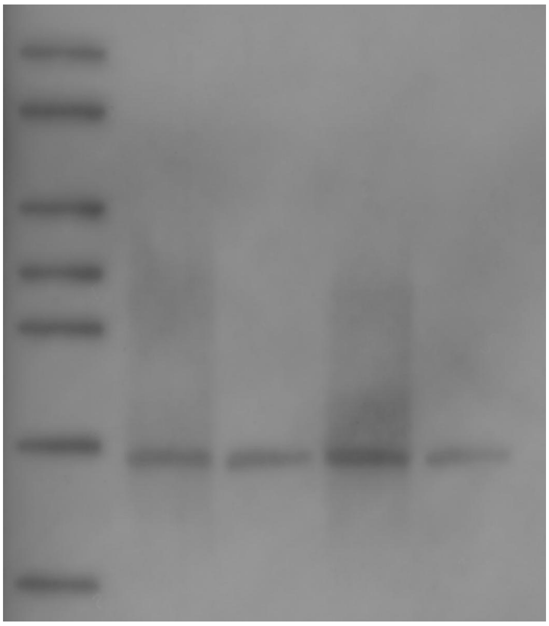

CD63

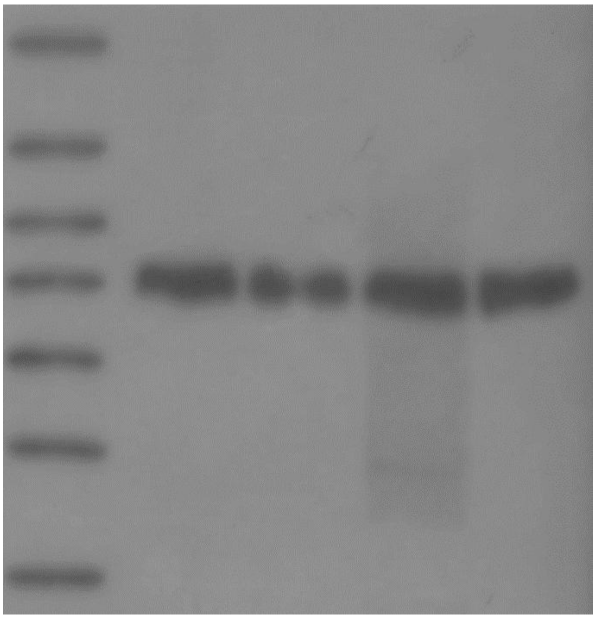

CD81

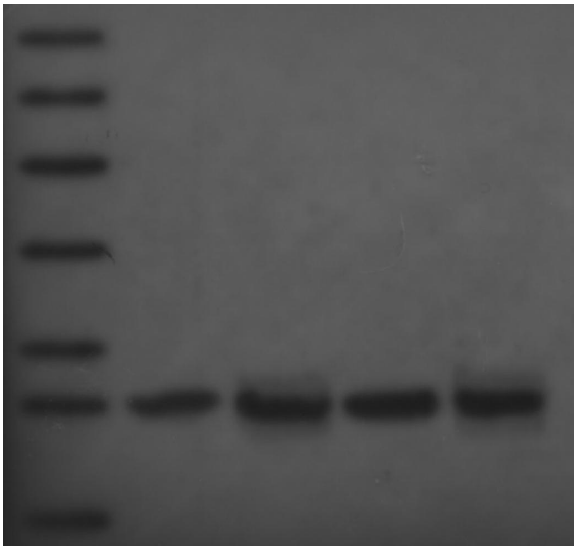

Calnexin

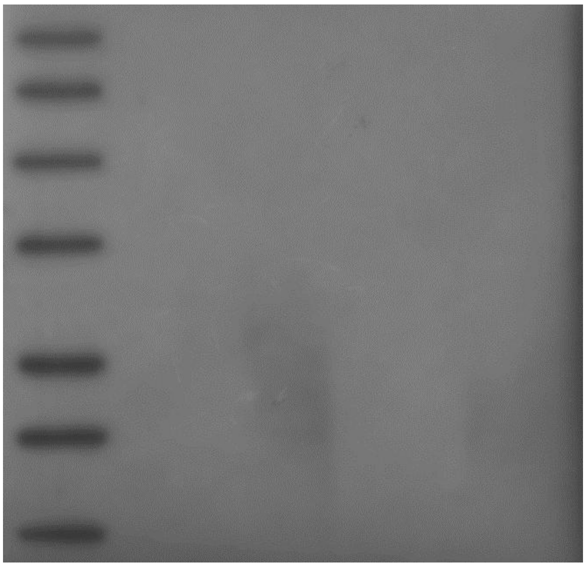

Fig 3E
GAL3

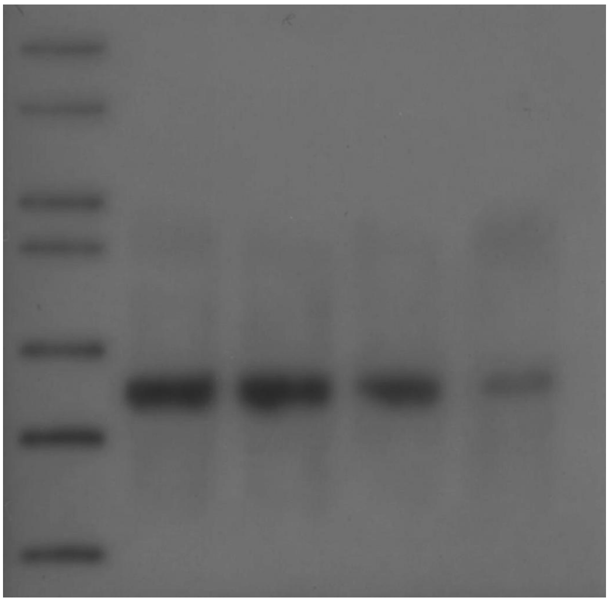

Fig 3F
GAL3

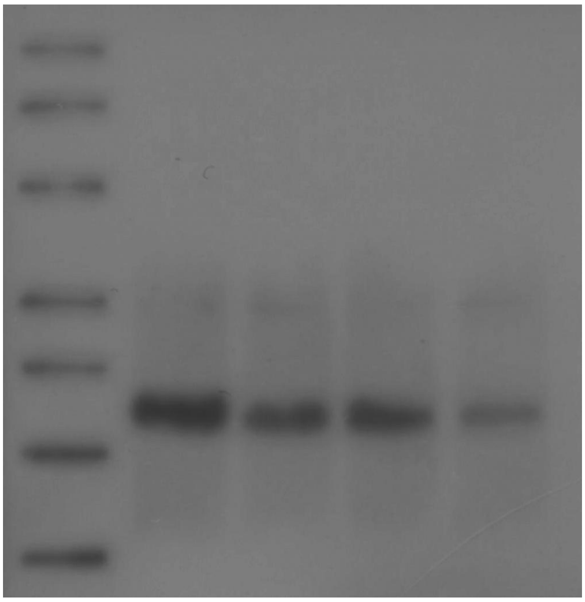

GAPDH

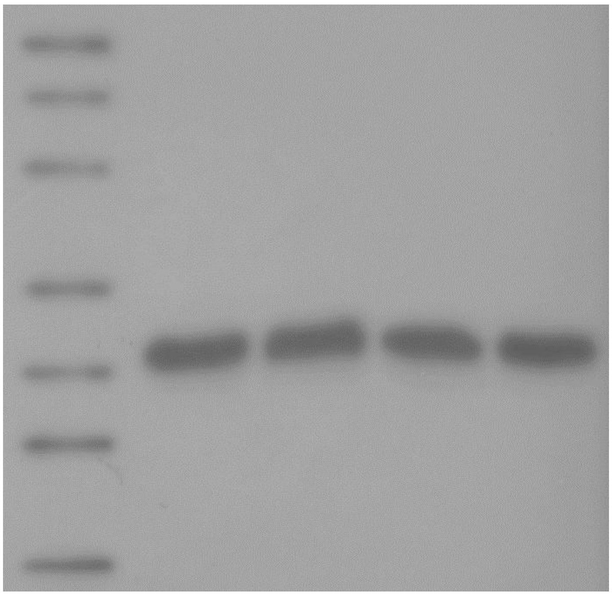

Fig 3G

GAL3

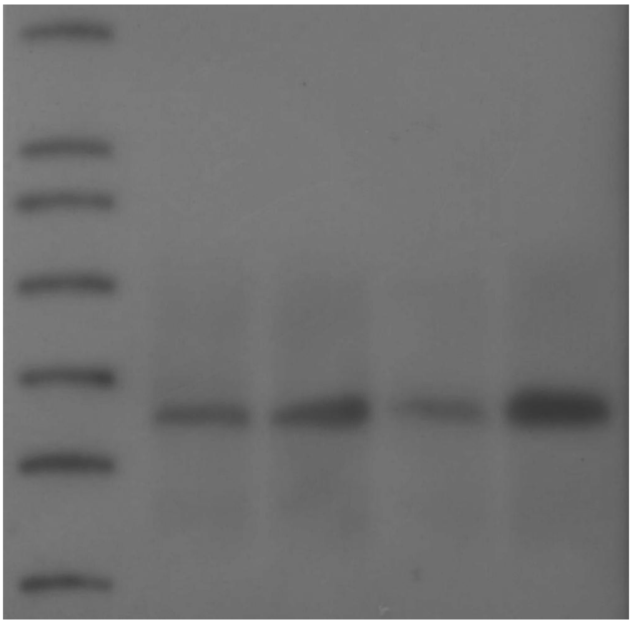

GAPDH

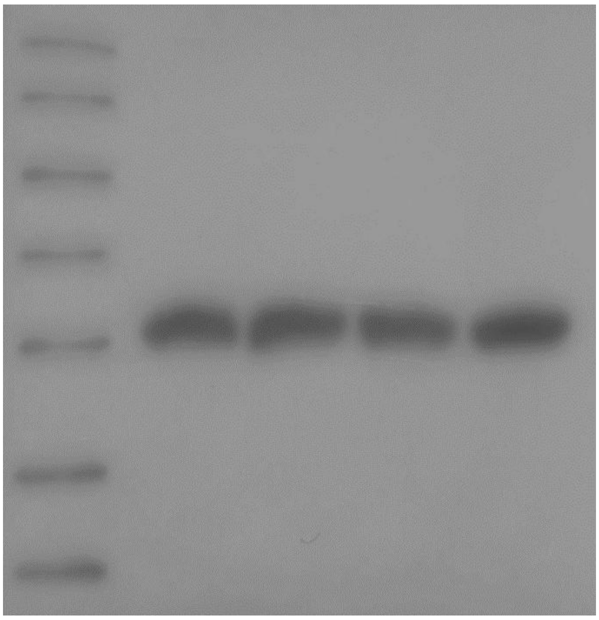

Fig 4A

β-catenin

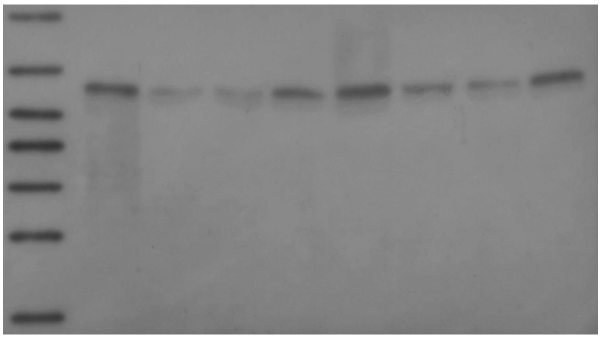

Histon H3

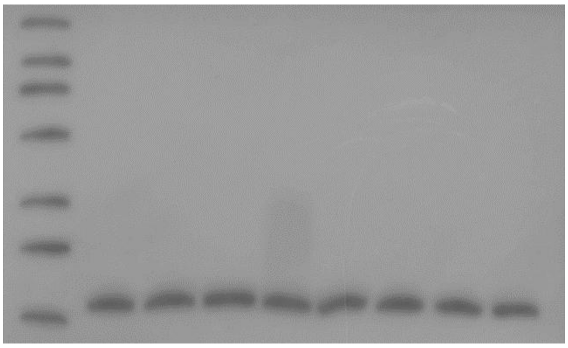

β-catenin

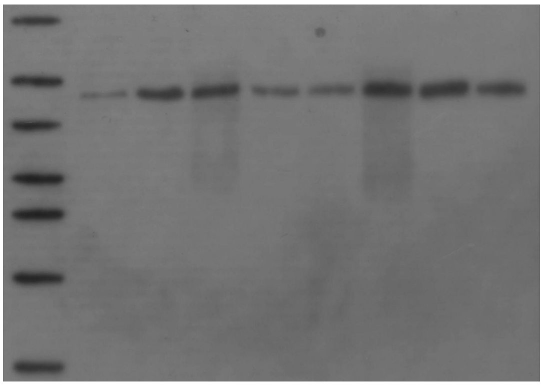

GAPDH

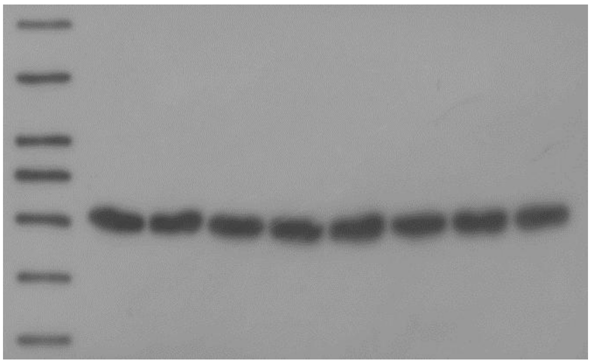

Fig 5E

β-catenin

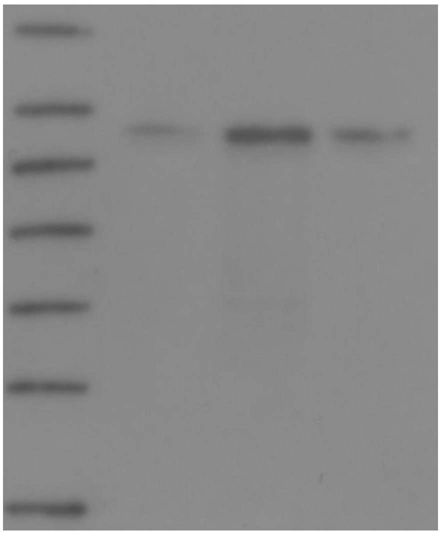

Histon H3

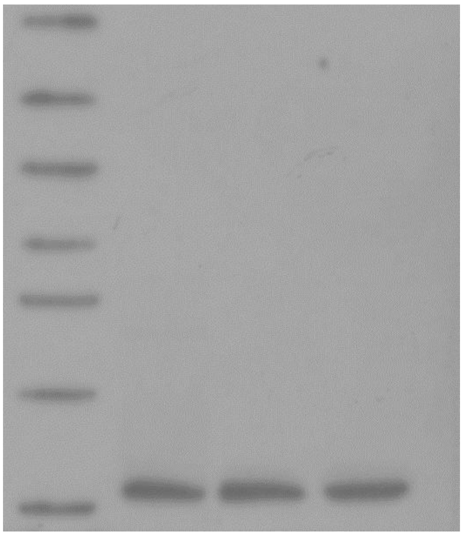

β-catenin

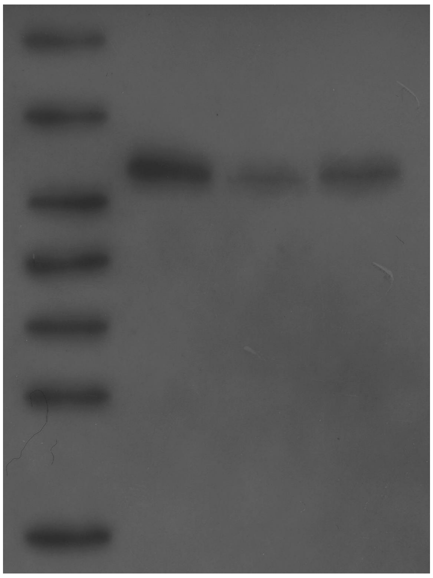

GAPDH

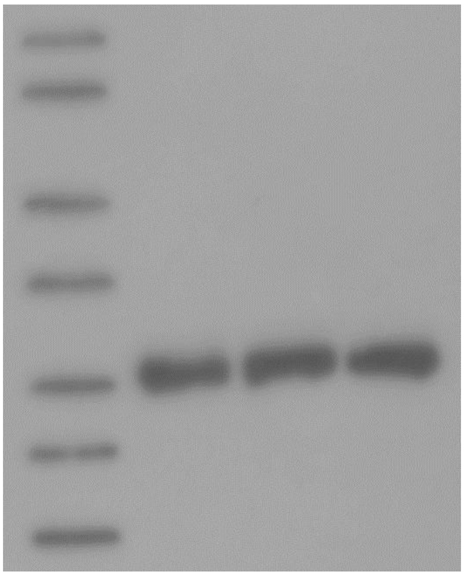

Supplement: Supplementary file 2 — Supplementary Material 2 [file 13048_2024_1430_MOESM2_ESM.pdf]
